# Label-free monitoring of tissue biochemistry following traumatic brain injury using Raman spectroscopy

**DOI:** 10.1039/c6an02238c

**Published:** 2016-11-28

**Authors:** Jakub Maciej Surmacki, Laura Ansel-Bollepalli, Francesca Pischiutta, Elisa R. Zanier, Ari Ercole, Sarah Elizabeth Bohndiek

**Affiliations:** a Department of Physics , University of Cambridge , JJ Thomson Avenue , Cambridge , CB3 0HE , UK . Email: js2238@cam.ac.uk ; Email: laura.bollepalli@cruk.cam.ac.uk ; Email: seb53@cam.ac.uk ; Tel: +44 (0)1223 769697 ; Tel: +44 (0)1223 337267; b Cancer Research UK Cambridge Institute , University of Cambridge , Robinson Way , Cambridge , CB2 0RE , UK . Tel: +44 (0)1223 769697 ; Tel: +44 (0)1223 337267; c Department of Neuroscience , IRCCS - Istituto de Ricerche Farmacologiche Mario Negri , Via G. La Masa 19 , 20156 Milano , Italy . Email: francesca.pischiutta@marionegri.it ; Email: elisa.zanier@marionegri.it ; Tel: +39 02 39 014 721 ; Tel: +39 02 39 014 204; d Division of Anaesthesia , University of Cambridge , Addenbrooke's Hospital , Hills Road , Cambridge , CB2 0QQ , UK . Email: ae105@cam.ac.uk ; Tel: +44 (0)1223 217889

## Abstract

Traumatic brain injury (TBI) constitutes a major cause of death and long-term disability. At present, we lack methods to non-invasively track tissue biochemistry and hence select appropriate interventions for patients. We hypothesized that detailed label-free vibrational chemical analysis of focal TBI could provide such information. We assessed the early spatial and temporal changes in tissue biochemistry that are associated with brain injury in mice. Numerous differences were observed in the spectra of the contusion core and pericontusional tissue between 2 and 7 days. For example, a strong signal from haem was seen in the contusion core at 2 days due to haemorrhage, which subsequently resolved. More importantly, elevated cholesterol levels were demonstrated by 7 days, which may be a marker of important cell repair processes. Principal component analysis revealed an early ‘acute’ component dominated by haemorrhage and a delayed component reflecting changes in protein and lipid composition. Notably we demonstrated changes in Raman signature with time even in the contralateral hemisphere when compared to sham control mice. Raman spectroscopy therefore shows promise as a probe that is sensitive to important pathobiological processes in TBI and could be applied in future both in the experimental setting, as well as in the clinic.

## Abstract 2

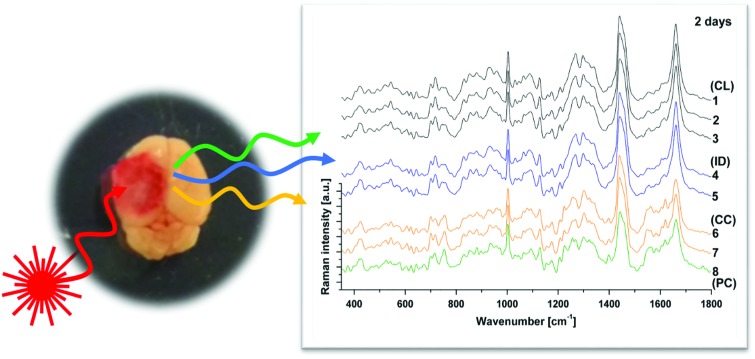
Acute tissue biochemical response to traumatic brain injury is revealed using Raman spectroscopy.

## Introduction

Traumatic brain injury (TBI) constitutes a major cause of death and long-term disability, including late cognitive deficits in survivors of even milder injuries. The societal costs of acute and chronic care, as well as from lost economic activity, for such patients is enormous. TBI is a complex disease whose pathogenesis continues for weeks after the initial injury. Progress towards improving outcome in patients has been limited because the complex, multifactorial pathobiology of TBI is not well understood; hence opportunities for therapeutic intervention are missed.

Studies to date indicate that TBI is a disease governed by the host response to the injury, which varies dramatically between patients and has a complex spatiotemporal profile. Some commonalities include cell damage and loss of axonal connectivity over the first 24 hours and neuronal phospholipid membrane breakdown, clinically evidenced by early elevation in extracellular glycerol concentrations.^1^ A variety of molecular processes, such as excitotoxicity/calcium influx, oxidative injury and inflammation, along with cellular changes in resident microglial cells and recruitment of macrophages, may continue to drive injury.^2^–^4^ These insights have been provided by traditional experimental techniques, such as immunohistochemistry that must be performed *ex vivo* and has specificity to predetermined epitopes only. Cerebral microdialysis can provide some chemical information *in vivo* but has poor spatial resolution and is only sensitive to small molecules and these must be present in the extracellular fluid. Non-invasive tools to track brain tissue biochemistry after injury remains an unmet need, which if met, could shed light on disease pathobiology and furthermore, assist clinically with translation of precise and timely interventions, such as neuroprotective therapies.^5^

Raman spectroscopy (RS) is a powerful label-free analytical technique that has been widely used to study tissue biochemistry, for example, profiling lipid, protein and nucleic acid composition.^6^–^8^ All twelve major and minor human brain lipids can be identified using RS at 785 nm.^9^ Previous work has revealed that RS can indicate brain tissue damage in response to both radiation^10^ and penetrating^11^ injuries. In the latter study, RS was able to delineate injured and healthy brain regions in a mouse model of motor cortex needle injury on the basis of reduced signal intensity at 1660 cm^–1^, attributed to amide I vibration of proteins and C=C stretching of lipids, and at 1002 cm^–1^, attributed to the ring breathing mode of phenylalanine in proteins.^11^ However, these data were acquired exclusively in the motor cortex and penetrating injury does not reflect the vast majority of TBI seen clinically. Furthermore, the study was conducted at a single time point immediately after injury, which does not provide crucial information on the evolution of the pathology.^2^–^4^

We hypothesized that RS could monitor the progression and resolution of brain injury over space and time in a clinically relevant model of focal TBI. To test this hypothesis, we studied for the first time using RS the spatial and temporal distribution of early changes in brain biochemistry after focal severe TBI modelled by controlled cortical impact in mice. We found that RS provides a sensitive readout of the evolution of tissue injury in our model.

## Experimental

### Animals

C57BL/6 mice (adult male mice, Envigo, Italy) were housed in a specific pathogen free vivarium at a constant temperature (21 ± 1 °C) with a 12 h light-dark cycle and *ad libitum* access to food and water. The IRCCS-Instituto di Ricerche Farmacologiche Mario Negri (IRFMN) adheres to the principles set out in the following laws, regulations, and policies governing the care and use of laboratory animals: Italian Governing Law (D.lgs 26/2014; Authorization no. 19/2008-A issued March 6, 2008 by Ministry of Health); Mario Negri Institutional Regulations and Policies providing internal authorization for persons conducting animal experiments (Quality Management System Certificate – UNI EN ISO 9001:2008 – Reg. No. 6121); the NIH Guide for the Care and Use of Laboratory Animals (2011 edition) and EU directives and guidelines (EEC Council Directive 2010/63/UE). The Statement of Compliance (Assurance) with the Public Health Service (PHS) Policy on Human Care and Use of Laboratory Animals has been recently reviewed (9/9/2014) and will expire on September 30, 2019 (Animal Welfare Assurance #A5023-01). All efforts were made to minimize animal suffering and to reduce the number of animals used.

### Experimental traumatic brain injury

Adult male mice (11 week old) were anesthetized with isoflurane inhalation (induction: 3%, maintenance: 1.5%) in an N_2_O/O_2_ (70%/30%) mixture and placed in a stereotaxic frame. Rectal temperature was maintained at 37 °C. Mice were then subjected to craniectomy followed by induction of controlled cortical impact brain injury as previously described.^12^–^15^ The injury was induced using a 3 mm rigid impactor driven by a pneumatic piston rigidly mounted at an angle of 20° from the vertical plane and applied vertically to the exposed dura mater, between bregma and lambda, over the left parieto-temporal cortex (antero-posteriority: –2.5 mm, laterality: –2.5 mm), at impactor velocity of 5 ms^–1^ and deformation depth of 1 mm. The craniotomy was then covered with a cranioplasty and the scalp sutured. Sham (control)-operated mice received identical anesthesia and surgery without brain injury.

Mice were sacrificed at 2 or 7 days after TBI (*n* = 3 per time point) under deep anesthesia (ketamine 30 mg/medetomidine 0.3 mg). The brains were then removed, washed once with phosphate buffered saline (PBS) then frozen immediately into liquid nitrogen and stored at –80 °C. Before commencing Raman analysis brains were thawed for 20 min at room temperature.

### Reference chemicals

Cholesterol (Acros Organics, CAS 57-88-5), cholesteryl nonanoate (Eastman Organic Chemicals, no. 9669), cholesteryl oleyl carbonate (Eastman Organic Chemicals, no. 10053), intralipid (Sigma-Aldrich, no. I141) and albumin from bovine serum (Sigma-Aldrich, no. A7906) were investigated.

### Raman data acquisition and analysis

Raman spectroscopy was performed with a confocal Alpha 300M+ Raman microscope (WITec GmbH, Ulm, Germany) equipped with a 785 nm single mode diode laser (XTRA II; Toptica Photonics Inc., USA), a 300 mm triple grating imaging spectrometer (Acton SpectraPro SP-2300; Princeton Instruments Inc., USA) with 600 g mm^–1^ grating (BLZ = 750 nm), a thermoelectrically cooled CCD camera (DU401A-BV; Andor, Ireland) and 20× objective (LD Plan-Neofluar; Zeiss, Germany). Although here we are collecting data using a confocal microscope, it should be noted that previous studies have found exciting tissue at 785 nm can yield Raman signals from 5–10 mm of depth, depending on the optical properties of the tissue.^16^

Whole brain samples were placed on a 25 mm round microscope coverslip in a petri dish and illuminated with 35 mW at 785 nm. Single Raman spectra were collected with 1 s integration time and 10 accumulations. At each position interrogated (as indicated on the figures), at least 10 spectra were collected along a line scan up to 70 μm length. Total numbers of recorded Raman spectra used in the analysis are: contralateral (CL; *n*(2 days) = 330, *n*(7 days) = 330, *n*(control) = 560); pericontusional (PC; *n*(2 days) = 330, *n*(7 days) = 180, *n*(control) = 240); contusion core (CC; *n*(2 days) = 140, *n*(7 days) = 240, *n*(control) = 360) and ipsilateral tissue distant from the contusion core (ID; *n*(2 days) = 120, *n*(7 days) = 120, *n*(control) = 200).

Data processing was performed using Project Plus Four 4.1 (WITec), Origin 2016 (OriginLab) and MATLAB with PLS-Toolbox (Mathworks). All Raman spectra were cosmic ray and baseline corrected then normalized to the area-under-curve. Statistical significance was analysed using two-way ANOVA, followed by Tukey's multiple comparison test. *p* < 0.05 was considered statistically significant.

## Results

**Fig. 1:**
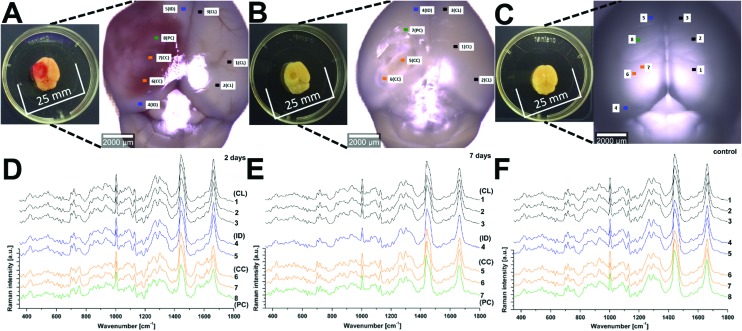
Raman spectroscopy reveals changes in tissue biochemistry following traumatic brain injury (TBI). Bright field microscopy composites of example murine brains at 2 days (a) and 7 days (b) after TBI, as well as control (c) (Inset photographs show sample preparation). Average Raman spectra shown in (d), (e) and (f) were acquired from the areas indicated in matching colours in the photos (a), (b) and (c). CL = contralateral; ID = ipsilateral distant from contusion core; PC = pericontusional tissue; and CC = contusion core. Spectra were acquired at 785 nm, with 1 s exposure and 10 accumulations; the average is performed over 30 separate spatial points.

**Fig. 2:**
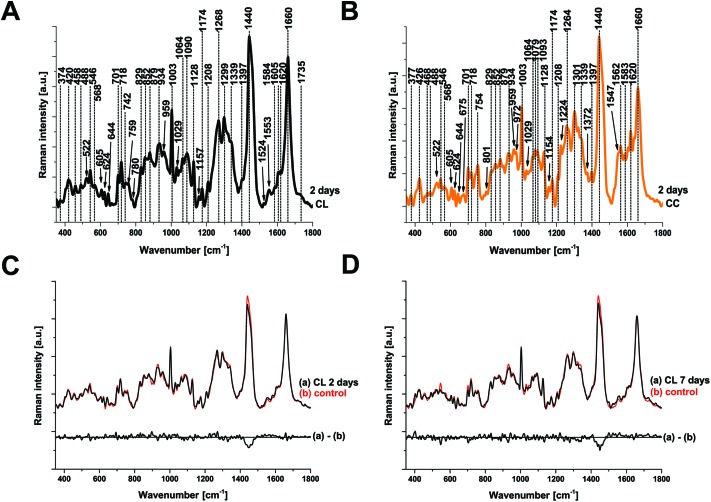
Peak identification in average Raman spectra and comparison of the average Raman spectra of contralateral with sham control at 2 and 7 days after TBI. (a) Average spectrum of the contralateral (CL) and (b) the contusion core (CC). No additional peaks were identified at 7 days, so only data from 2 days are shown for clarity. Comparison of CL to sham brains was made at 2 (c) and 7 (d) days after injury. Spectra were acquired at 785 nm, with 1 s exposure and 10 accumulations; the average is performed over 30 separate spatial points.

Raman spectroscopy (RS) was performed at a range of spatial locations on the excised brains at 2 (Fig. 1A) and 7 (Fig. 1B) days after TBI, or in sham control brains (Fig. 1C). The spatial locations are identified as: contralateral (CL); ipsilateral tissue, distant from the contusion core (ID); pericontusional (PC); and contusion core (CC). The injury was delivered to the left parietotemporal cortex, so the right hemisphere is contralateral. Significant discoloration in the injured site can be observed in the sample at 2 days of TBI as a result of mechanical damage to the tissue and associated bleeding (Fig. 1A). The spatial and temporal distribution of the RS data (Fig. 1D–F) shows dominant spectral contributions from lipids and proteins as expected from tissue. Example spectra from 2 days after TBI are annotated in detail in Fig. 2; no additional peaks were identified at 7 days, so only data from 2 days are annotated for clarity. Comparing qualitatively the contralateral hemisphere with the contusion core (Fig. 2A and B) at 2 days after TBI, we can see additional strong bands at ∼754, 1224, 1547, 1562, 1620 cm^–1^ from haemoglobin and decreased intensity of the bands at around 718, 1264, 1440 and 1660 cm^–1^ from lipids. We also recorded spectra at the coordinates of the contusion core in sham (control) mice. Based on their similarity to the spectra of the contralateral hemisphere at 2 and 7 days after TBI (Fig. 2C and D), for all further analysis we compared contusion, pericontusional and ipsilateral distant data to the contralateral side of the same brain.

**Fig. 3:**
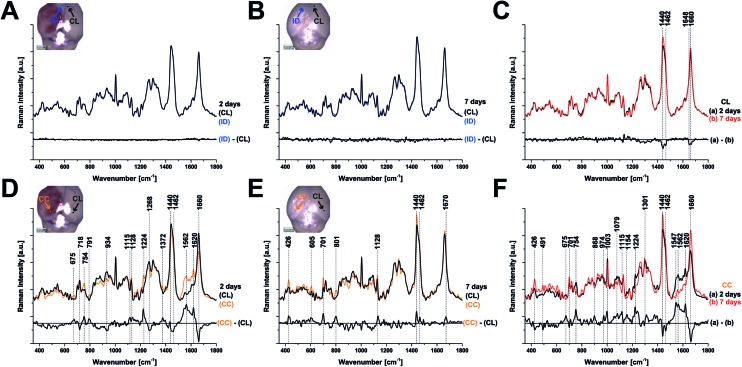
Comparison of the average Raman spectra at 2 and 7 days after TBI. Each panel also shows a difference spectrum for clarity. (a, b) Contralateral (CL) compared with ipsilateral distant tissue (ID) are identical at either 2 or 7 days, however, a difference in the peaks around 1440 and 1660 cm^–1^ (c) arises in CL tissue over time (between 2 and 7 days) showing that there are global changes in chemistry remote from the contusion even in apparently uninjured tissue. At 2 days after TBI, the spectra from the contusion core (CC) and CL are markedly different (d) and these differences evolve rather than normalise by day 7 (e, f). Spectra were acquired at 785 nm, with 1 s exposure and 10 accumulations; the average is performed over 30 separate points.

To examine the changes recorded by RS in more detail, we computed the differences between the average spectra across all mice at each spatial location and time point (Fig. 3). No significant difference was observed between the CL hemisphere and ID measurements made at ‘uninjured’ tissue away from the contusion core (identified qualitatively from the bright field images) either at 2 or 7 days (Fig. 3A and B). Small differences are apparent when comparing contralateral control tissue between 2 days and 7 days, associated with the peaks at 1140, 1462, 1648, and 1660 cm^–1^ (Fig. 3C). Striking differences arise between the contralateral controls and the contusion core at 2 days (Fig. 3D). Some of these resolve with time, in particular the spectral shoulder between the peaks at 1562 and 1620 cm^–1^, which we ascribe to resonance excitation of haemoglobin,^17^ but the spectral signatures remain very different compared to contralateral also at 7 days (Fig. 3E). Comparing the contusion core between 2 days and 7 days (Fig. 3F), interesting spectral differences can be noted in bands at 426, 701, 801, 1128, 1301, 1440, 1462 and 1660 cm^–1^, associated with lipids. Peaks at 426, 675, 754, 1003, 1224, 1547, 1562, 1620 cm^–1^ are likely due to resonance excitation of haemoglobin;^17^ the extensive area of bleeding present at 2 days after TBI has largely resolved by 7 days resulting in a decreasing signal of haemoglobin (Fig. 1A and B). Pericontusional tissue showed similar changes to the contusion core.

**Fig. 4:**
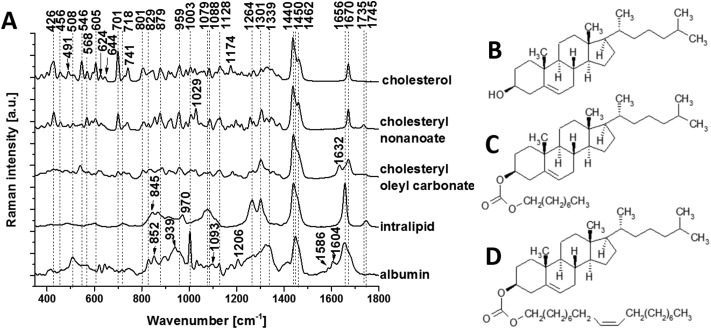
Raman spectral characterization of lipids. (a) Average Raman spectra of the cholesterol (b), cholesteryl nonanoate (c), cholesteryl oleyl carbonate (d) and intralipid (no structure shown as mixed lipids). Protein albumin is also shown for reference. Spectra were acquired at 785 nm, with 1 s exposure and 10 accumulations at 175 mw; the average is performed over 10 separate spatial points.

To isolate the origin of the changing lipid signal observed, we acquired average Raman spectra (Fig. 4A) of cholesterol (Fig. 4B), cholesteryl nonanoate (Fig. 4C), cholesteryl oleyl carbonate (Fig. 4D), as well as a mixed lipid solution (intralipid) and protein albumin. The most intense bands from cholesterol are at 426, 546, 605, 701, 741, 1128, 1440, 1462 and 1670 cm^–1^; the band at 1440 cm^–1^ can be assigned to CH deformation and 1670 cm^–1^ to C=C stretch vibrations.^9^ Additional bands at 1735 cm^–1^ (ester group) as well as 1029 cm^–1^ and 1301 cm^–1^ (fatty acid chains) are observed in the spectra of cholesterol derivatives. Characteristic bands of intralipid (phospholipid stabilized soybean oil) are at 718, 845, 879, 970, 1064, 1079, 1264, 1301, 1440, 1656 and 1745 cm^–1^. Symmetric and antisymmetric stretch vibrations of the choline group N^+^(CH_3_)_3_ in phospholipid^9^ can be found at 718 and 879 cm^–1^. Albumin is the most common α-helical protein with the Raman bands at 508, 852, 1003, 1093, 1206, 1339, 1450, 1586, 1604 and 1656 cm^–1^. Evidence of these Raman bands from cholesterol, cholesterol derivatives, phospholipids and protein are observed in our RS data from the brain samples.

**Fig. 5:**
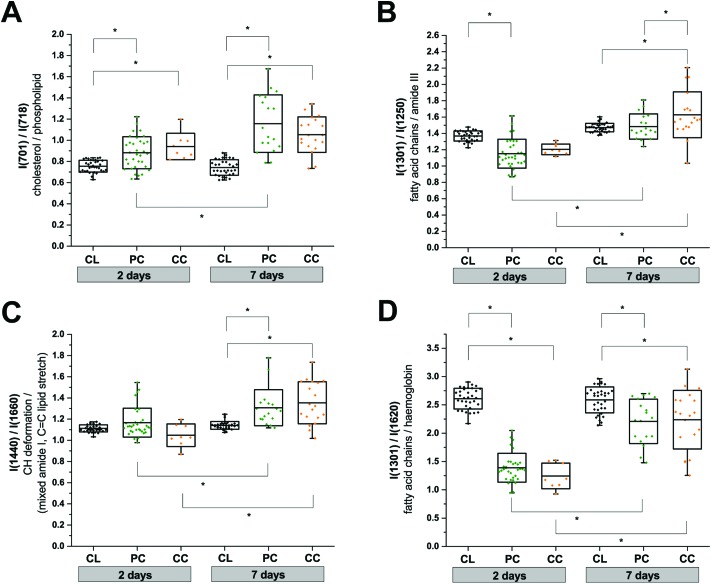
Two-way anova analysis of Raman peak intensity ratios. (a) *I*(701)/*I*(718), (b) *I*(1301)/*I*(1250), (c) *I*(1440)/*I*(1660), and (d) *I*(1301)/*I*(1620). Replicates used in the analysis are: contralateral (CL; *n*(2 days) = 33, *n*(7 days) = 33); pericontusional tissue (PC; *n*(2 days) = 33, *n*(7 days) = 18) and contusion core (CC; *n*(2 days) = 8, *n*(7 days) = 19). Box plots indicate means ± sd; *p < 0.05 was considered as statistically significant. Spectra were acquired at 785 nm, with 1 s exposure and 10 accumulations; the average is performed over 10 separate spatial points.

We used the knowledge of these peak identifications to guide our statistical analysis to assess the most significant spatial and temporal changes in the RS data. Changes in lipid and protein metabolism are of particular interest biologically after TBI in relation to cell damage and inflammatory processes. Unfortunately, many lipid and protein Raman signals overlap which makes a clean interpretation difficult. Nevertheless, in an attempt to obtain some mechanistic insight, we performed two-way ANOVA tests using the extracted peak intensity ratios for *I*(701)/*I*(718) (cholesterol^9^/phospholipid;^7^,^9^ Fig. 5A), *I*(1301)/*I*(1250) (mixed fatty acid chains^7^–^10^ and amide III protein;^8^,^10^ Fig. 5B), *I*(1440)/*I*(1660) (CH deformation^7^–^10^/mixed amide I protein^7^,^8^,^10^ and C=C stretching of lipids;^7^–^10^ Fig. 5C), and *I*(1301)/*I*(1620) (mixed amide III protein^8^,^10^ and fatty acid chains^7^–^10^/haemoglobin;^17^Fig. 5D). All metrics showed a statistically significant difference between the contralateral and pericontusional/contusional regions at 7 days after TBI; all except *I*(1440)/*I*(1660) also showed changes at 2 days after TBI. *I*(1301)/*I*(1250) also showed a significant difference between the pericontusional and contusional locations. Interestingly a large effect was observed in the ratio *I*(701)/*I*(718) demonstrating a significant relative increase in cholesterol and decrease in phospholipid contributions.

**Fig. 6:**
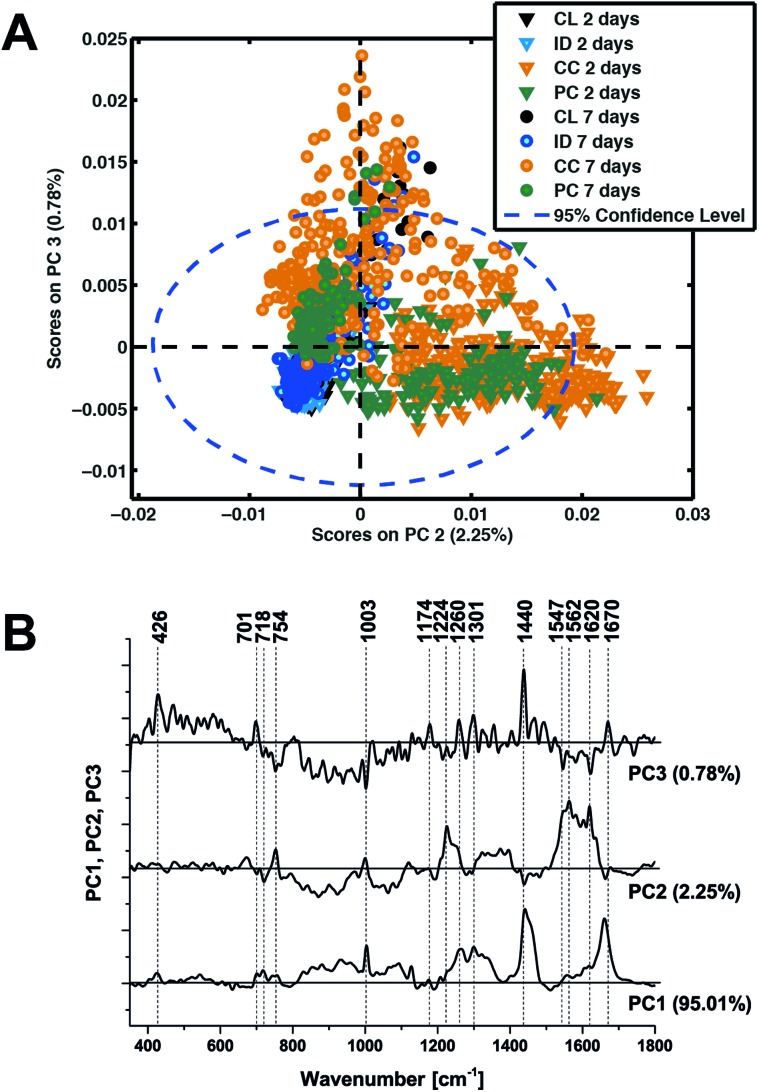
Principal components analysis of all Raman spectroscopy data. (a) Scatter plot of the score values of each single Raman spectrum for the second and third principal components from the murine brains 2 days (triangles) and 7 days (circles) after TBI: contralateral (CL, black), ipsilateral distant (ID, blue), contusion core (CC, orange) and pericontusional tissue (PC, green). (b) Loadings plot of principal components PC1, PC2 and PC3 indicating the peaks that contribute to these principal components.

In an attempt to determine a TBI ‘fingerprint’ we subsequently performed principal component analysis on the entire data set. The distribution of scores on principal components 2 and 3 (Fig. 6A) clearly distinguish the contusion and pericontusion regions from the distant contusional region and contralateral hemisphere, as well as the temporal evolution of the data at 2 and 7 days after TBI, based on the illustrated loadings (Fig. 6B). From Fig. 6A, principal component 2 is most effective at distinguishing injured and control tissue whereas component 3 discriminates better at 7 days. From Fig. 6B, PC2 is dominated by intense signals between 1547 and 1620 cm^–1^, representing resonance Raman shift from haemoglobin and so the acute phase is dominated by the contribution from bleeding at/near the contusion site. An important feature of PC3 the peaks at 701 cm^–1^ representing an increase in cholesterol signal with respect to the 718 cm^–1^ phospholipid peak.

## Discussion

RS is a powerful label-free analytical technique that has been widely used to study tissue biochemistry. Our results demonstrate that RS can track the evolution of TBI over the first 7 days following injury. While the contralateral and ipsilateral distant regions are similar at the two time points studied, the spectrum of the contusion core is strikingly different already at 2 days after injury and evolves further up to 7 days.

One striking spectral feature results from resonant excitation of haemoglobin in the pericontusional and contusion core regions, consistent with haemorrhage resulting from vascular disruption and bleeding at the lesion.^18^ Haemorrhagic conversion and contusion expansion is a potentially devastating pathology after TBI, which can lead to oxidative damage and cytotoxicity^19^ so it is particularly important that any non-invasive monitoring technique is sensitive to this process. The haemoglobin signals are no longer apparent by 7 days; the macroscopic appearance of the tissue is consistent with the phagocytic clearance of erythrocytes and endocytosis of haemoglobin and haem by macrophages/microglia.^20^

A second striking spectral feature appears in the pronounced peaks at 701 cm^–1^, assigned to cholesterol and 718 cm^–1^, assigned to phospholipid. Our normalised spectra show a relative increase of cholesterol signal and decrease in phospholipid in the contusion core compared to the contralateral tissue and over time. The relative change at 701/718 cm^–1^ provides strong evidence of a change in lipid chemistry in the pericontusional tissue and contusion core at 2 days that remains at 7 days. Cholesterol is known to be critical for cellular repair, in neurodegenerative diseases^21^ and the apolipoproteins involved in cholesterol transport are up-regulated at 4 days after TBI.^22^ Furthermore, the ε4 allele of apolipoprotein E has been associated with worse outcomes after TBI^23^ and increased risk of post-TBI dementia.^24^ Interestingly, local cholesterol exposure may alter the accessibility of amyloid precursor protein (APP) to β-secretase (Bace1), an enzyme responsible for the cleavage of APP to Amyloid-β (Aβ).^24^,^25^ The accumulation of Aβ is recognised as a pathognomonic mechanism in Alzheimer's and can also be seen within hours of TBI.^26^ These considerations suggest that RS could in future provide a probe of these critical mechanisms in TBI pathogenesis.

Another spectral feature characteristic of the TBI appeared at 1440/1660 cm^–1^. This feature has been used as a marker of the relative concentration of β-sheet protein (amide I peak at 1660 cm^–1^) – a hallmark of amyloid Aβ.^27^ While it is hard to disentangle changes in lipid/protein content in regions of the spectrum around 1440 and 1660 cm^–1^, RS clearly identifies changes in lipid content with respect to protein, consistent with previous findings.^11^ A final feature of note is the increased heterogeneity of measurements between the contusion and pericontusional tissue reflecting the spatial heterogeneity and temporal evolution of the contusion.^3^,^28^ Further studies combining RS and conventional immunohistochemical analysis will allow us to understand the contribution of cell population activation state to the RS brain biochemistry.

There are some limitations to this study. Small differences were observed in the 1440/1462 cm^–1^ and 1648/1660 cm^–1^ protein/lipid complex between the spectra recorded in sham mice and contralateral region of injured mice, as well as between the contralateral region at 2 and 7 days. Further work is needed to determine which underlying biological changes could impact brain biochemistry far from the contusion core. Examples include excitotoxicity, inflammation, blood brain barrier breakdown, oedema or intracranial pressure. We are unable, however, from our present data to characterise the cellular population in the volume being probed; correlation with histology and immunohistochemistry over space and time is required to fully understand the pathological underpinnings of our findings. Nonetheless, since RS could be applied *in vivo*, these findings are promising for tracking the temporal and spatial evolution brain tissue biochemistry after injury.

## Conclusions

We have shown that RS is a sensitive and reproducible label-free technique for monitoring response to TBI. Uniquely, we have used a clinical relevant mouse model of parietotemporal contusion and studied both spatial and temporal changes in the RS TBI signature. Changes in cholesterol (peak at 701 and 1670 cm^–1^), phospholipid (718 and 1301 cm^–1^) and protein (1250 and 1660 cm^–1^) signals as well as a strong (resonance) signal from haem (1547 and 1620 cm^–1^) are all potentially biologically important biomarkers of interest when considering the pathological evolution of TBI. RS therefore shows promise both as an experimental tool for the study of TBI as well as a possible clinical monitor, allowing therapeutic interventions to be personalised and delivered at time points where particular pathobiological mechanisms are dominant. Future work is needed to clearly relate the spatiotemporal spectroscopic changes to underlying pathobiology.
